# A Novel Tangential Electric-Field Sensor Based on Electric Dipole and Integrated Balun for the Near-Field Measurement Covering GPS Band

**DOI:** 10.3390/s19091970

**Published:** 2019-04-26

**Authors:** Jianwei Wang, Zhaowen Yan, Wei Liu, Donglin Su, Xin Yan

**Affiliations:** 1 School of Electronic and Information Engineering, Beihang University, Beiijng 100191, China; by1702120@buaa.edu.cn (J.W.); by1602164@buaa.edu.cn (W.L.); sdl@buaa.edu.cn (D.S.); 2 EMC Laboratory, Missouri University of Science and Technology, Rolla, MO 65409, USA; yx9n9@mst.edu

**Keywords:** near-field, electric dipole, tangential electric-field sensor, balun

## Abstract

This paper presents a novel tangential electric-field sensor with an embedded integrated balun for sensing up a tangential electric field over a circuit surface in the near-field measurements covering the GPS band. The integrated balun is embedded into the sensor to transform the differential voltage induced by the electric dipole into the single output voltage. The measurement system with a high mechanical resolution for the characterizations and tests of the sensor is detailed in this paper. The frequency response of the sensor characterized by a microstrip line from 1.35 GHz to 1.85 GHz (covering the GPS band) is rather flat. The rejection to the magnetic field of the sensor is up to 20.1 dB. The applications and validations of the sensor are conducted through passive/active circuit measurements.

## 1. Introduction

Most modern consumer electronics are more portable and more multifunctional. The electronic modules are distributed compactly in a specific room to achieve more portable and more reduced-sized consumer electronics. Unfortunately, compact distributions of electric modules increase the possibilities of electromagnetic compatibility (EMC) issues of consumer electronics. EMC issues must be taken into consideration before or in the design stage in order to ensure the operational robustness and reliability of consumer electronics [1].

The designs of EMC are increasingly challenging for engineers who want to make the field distribution over a circuit surface visible and find out which point or area is more strongly radiated. The accurate circuital and electromagnetic models have been established to evaluate these direct conducted and radiated emissions [2]. Near-field probes, such as electromagnetic sensors, have been widely applied to image surface [3], image microwave properties of materials [4], detecting surface cracks [5], measuring noise on a printed circuit board (PCB) [6], and measuring a dielectric constant [7,8]. The near-field data is obtained through near-field scanning, which is widely used to map the electromagnetic-field distribution over a specific surface above the device under test (DUT). The electromagnetic sensors are the sensing and picking-up elements in the near-field scanning. An electrically small loop, used as the sensing part for the magnetic-field sensor, can respond positively to the magnetic-field component oriented normally to the surface of the loop [9,10,11]. An open-ended monopole can be employed to pick up the normal electric field [12,13,14,15], while the electrically small electric dipole is utilized to detect the tangential electric field [16,17,18] over a surface above the planar circuit. The electromagnetic sensors can effectively sense electromagnetic fields so that there has been increasing attention given to developing high-performance electric-/magnetic-field sensors, such as extending the bandwidth [19,20,21], improving the spatial resolution [22,23,24] and enhancing the sensitivity [25,26,27,28]. Nowadays, there exists a situation where the reconstruction of the interference source based on the dipole moments needs to detect the tangential electric field [29,30]. In this case, engineers want to know how the tangential electric field distributes and what its exact values are.

In this paper, a tangential electric-field sensor covering the GPS band for EMC applications is proposed, fabricated, calibrated and validated through the measurements of passive and active circuits. The voltage induced by the sensing part of the sensor is in the differential mode. Thus, an integrated balun based on the power divider and phase shifter is designed to transform the differential-mode voltage to the single-end voltage. The measurement system is also presented. The performances of the sensor, such as the selectivity, sensitivity and rejection to the magnetic field, are investigated. The abilities to capture the tangential electric field of the sensor are validated through an HFSS simulation (a full-wave commercial software), as well as measurements of the passive and active circuits.

This paper is structured as follows. Section 2.1 introduces the reception mechanism of the electric dipole to clearly show the behavior of the tangential electric-field sensor when sensing a tangential electric-field component. After introducing the reception mechanism of the tangential electric-field sensor, the designs of the sensor are detailed in Section 2.2, including the structure of the sensor and the design of the integrated balun. In order to reduce the measurement time and improve the test efficiency, a measurement system is developed, and its details are presented in Section 3.1. The performances of the sensor are investigated in Section 3.2, Section 3.3, Section 3.4, and Section 3.5. The applications and validations are conducted through the passive and active circuits in Section 4. Finally, the conclusions are drawn in Section 5.

## 2. Theory and Methods

### 2.1. Reception Mechanism of the Sensor

**Figure 1:**
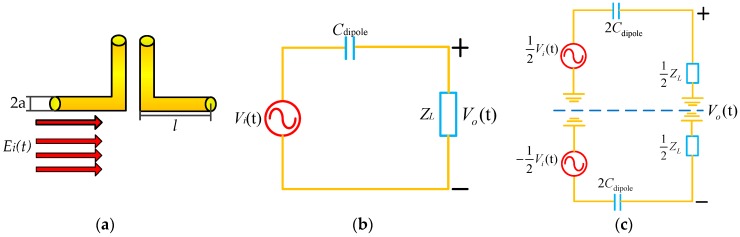
(**a**) Physical electric dipole. (**b**) Thevinin’s equivalent circuit model of an electric dipole when picking up a tangential electric-field component; (**b**) is the equivalent circuital form of (**c**) after applying odd/even-circuit analysis method.

The sensing part of the tangential electric-field sensor is an electrically small electric dipole. The reception mechanism of a small electric dipole (shown in Figure 1a) for picking up the tangential electric field, can be clearly explained through a Thevinin’s equivalent circuit model [31,32], shown in Figure 1b. The electric dipole can be modeled as a voltage in a series of capacitance $C_{\mathrm{dipole}}$. According to the odd/even-circuit analysis method, the Thevinin’s equivalent circuit model is transformed into the one shown in Figure 1c. $V_{i}\left(t\right)$ means the voltage sensed by the electric dipole when picking up the tangential electric-field component. The sensed voltage is proportional to the incident electric field strength ($E_{i}\left(t\right)$), which is parallel to the orientation of the electric dipole arm. The induced voltage is determined by the following equations [32,33].

(1) $$V_{i}(t)=h_{e}E_{i}(t)$$

(2) $$h_{e}=\frac{l\ln\left(\frac{4l^{2}}{a^{2}e}\right)}{\ln\left(\frac{16l^{2}}{a^{2}e^{2}}\right)}$$

where $h_{e}$ represents the antenna effective length; $E_{i}(t)$ represents the incident electric field strength; l means the half dimensional length of the electric dipole arm; a is the radius of the electric dipole arm; and e is the Euler number. It can be known from Equations (1) and (2) that the output voltage of the tangential electric-field sensor is proportional to the half-length of the electric dipole arm. The extension of the length of the electric dipole is one method to enhance the sensitivity of the tangential electric-field sensor. However, the extension of the length of the electric dipole arm will deteriorate the spatial resolution of the tangential electric-field sensor [33]. A well-working electromagnetic sensor should be the trade-off between the sensitivity and spatial resolution. The sensitivity is proportional to the length of the electric dipole arm, while the spatial resolution deteriorates with the increase of the length of the electric dipole arm. Reducing the sensitivity can improve the spatial resolution so that the sensor is able to detect a fine interference source. The reduced sensitivity can be compensated through cascading low noise amplifiers.

### 2.2. Design of the Tangential Electric-Dipole Sensor

#### 2.2.1. Integrated Balun

**Figure 2:**
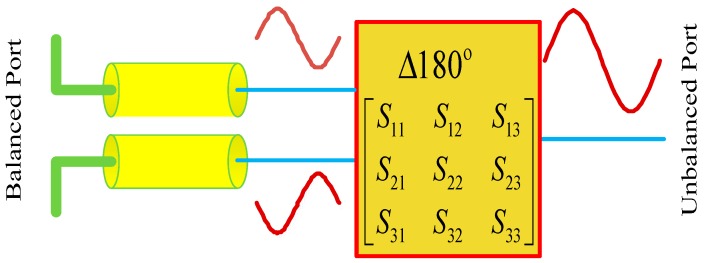
Diagram of the balun transferring the differential mode to the common mode.

As presented in Section 2.1, the electric dipole induces a differential-mode voltage in the series of a capacitance. Therefore, a balun is needed to achieve the transformation from the balanced to the unbalanced. Various types of baluns [34,35,36,37,38,39] have been designed to convert a balanced signal into two unbalanced signals with an equal amplitude and inverse phase. It should be noted that comparing the advantages of these baluns and then finding out the optimal one is not the focus of this paper. Figure 2 shows the mechanism of the transformation from the balanced port to the unbalanced port. In this paper, a balun with a center frequency of 1.575 GHz, based on a power divider and 180-degree phase shifter, is used to achieve the transformation from the differential mode to the common mode.

**Figure 3:**
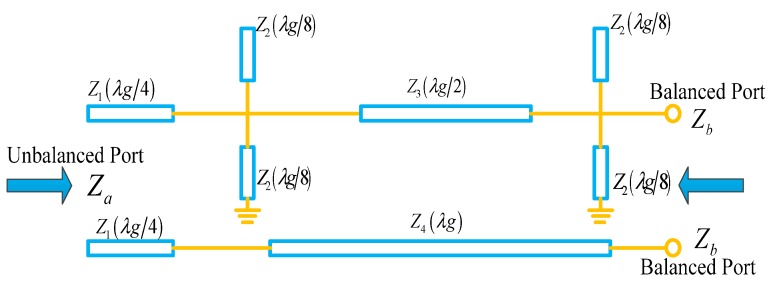
Equivalent circuit diagram of the balun.

**Figure 4:**
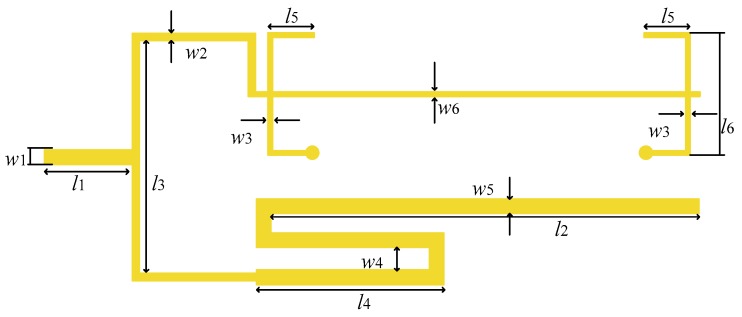
Layout of the designed balun.

From a previous study [40], the equivalent circuit of the balun based on the power divider and 180-degree phase shifter is shown in Figure 3. In order to simplify the design process, the case of the transformation from 50 Ω to 50 Ω for the balun is only considered, i.e., the unbalanced port impedance and the balanced port impedance are both 50 Ω. According to the studies in [39,40], the characteristic impedances of the transmission lines marked in Figure 3 are obtained, i.e., $Z_{1}=70.7\;\Omega$, $Z_{2}=63.5\;\Omega$, $Z_{3}=80.5\;\Omega$, and $Z_{4}=50\;\Omega$.The center frequency of the designed balun is 1.575 GHz. The layout of the balun with the physical dimensions is presented in Figure 4. One-lambda strip-line is folded to reduce the longitudinal length of the sensor. The physical dimensions of the designed balun are detailed in Table 1.

#### 2.2.2. Structure of the Sensor

**Figure 5:**
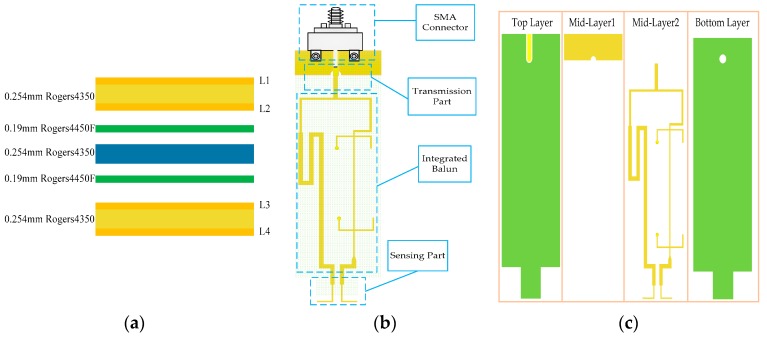
Detailed structure of the tangential electric-field sensor. (**a**) Stack-up; (**b**) overall top view; and (**c**) top view of each layer, to clearly exhibit the internal connections of the tangential electric-field sensor.

**Figure 6:**
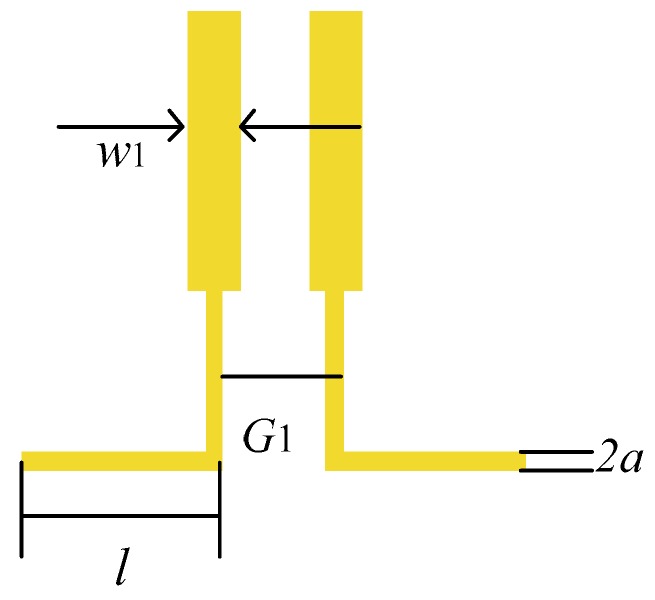
Electric dipole used as the sensing part of the tangential electric-field sensor. l = 4 mm. $G_{1}$ = 0.65 mm. $w_{1}$ = 0.35 mm. a = 0.05 mm.

The sensor for sensing the tangential electric field is proposed based on the PCB process. Figure 5 illustrates the detailed structure of the tangential electric-field sensor. The adopted PCB stack-up of the tangential electric-field sensor is presented in Figure 5a; its substrate is a high-performance Rogers material with a stable dielectric constant and low loss tangent. The thickness of the stack-up, consisting of three layers of 0.254 mm thick Rogers4350B (ε_r_ = 3.66) and two layers of 0.19 mm thick Rogers4450F (ε_r_ = 3.58), is 1.142 mm. Figure 5b shows the overall view of the designed tangential electric-field sensor that is composed of a sensing part, integrated balun, transmission part and sub-miniature-A (SMA) connector. The planar expanded structure of the tangential electric-field sensor is shown in Figure 5c in order to exhibit the internal connections clearly. The electric dipole depicted in Figure 6 is used as the sensing part for the sensor to sense the tangential electric field. The gap between the two electric dipole arms is 0.65 mm, and the length of each electric dipole arm is 4 mm, respectively. The copper planes on the top layer and bottom layer provide the shielding for the external field and a current return path. The electric dipole and integrated balun are cascaded in sequence, and routed on the third layer. Since the signal line transmitting of the induced voltage is on the third layer, a conductor-backed coplanar waveguide (CB-CPW) is designed to transport the voltage signal to the external port. The trace of the CB-CPW, whose referenced impedance plane is on the second layer, is routed on the top layer. The characteristic impedance of the CB-CPW trace is 50 Ω, and its width is 0.44 mm. The induced differential-mode voltage by the electric dipole is transformed into a common-mode voltage through the integrated balun.

## 3. Results and Discussions

### 3.1. Measurement System

**Figure 7:**
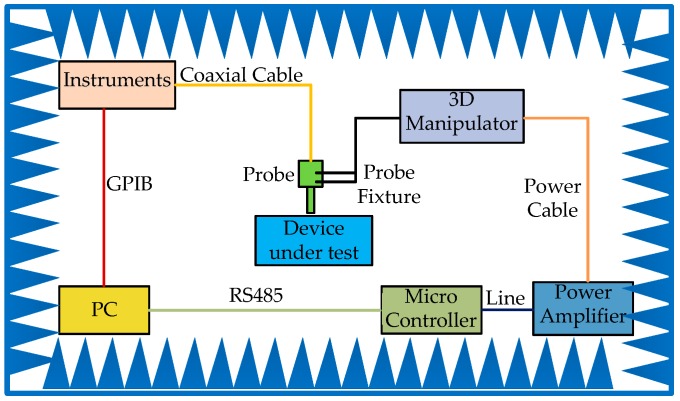
Block diagram of the measurement system.

In order to improve the measurement efficiency and save test time, a measurement system is developed. The block diagram of the measurement system is presented in Figure 7. Different measurement tasks are achieved by using the corresponding instruments. For the characterization of the flatness of the frequency response of the sensor, the instrument is the vector network analyzer (VNA); for the EMC measurement of the time domain, the instrument is the oscilloscope; for the EMC measurement of the frequency domain, the instrument is the spectrum analyzer or electromagnetic interference (EMI) receiver. The measurement software, named as the monitor software, is developed by Laboratory Virtual Instrument Engineering Workbench (LABVIEW); it is a friendly systems engineering software that is widely applied to measurements, tests and controls, with rapid access to hardware, and it is installed into the personal computer (PC). The monitor software on the PC is an interface with the client, which sends out instructions to the micro controller through RS485 communication, and which communicates with the instrument through a General Purpose Interface Bus (GPIB). The 3D manipulator consists of three mechanical arms with a respective stepper motor and one shockproof platform. After receiving the instructions, the micro controller will calculate, process, and then send out a series of low-level pulses containing the parameters of speed, steps, and motion direction to the motors on the mechanical arms. The low-level pulses are not high-power enough to drive the motor directly. Thus, these pulses are amplified by power amplifiers. The main parameters used to characterize the measurement system are summarized in Table 2. In order to reduce the development time, a commercial micro controller with a high stability and high precision is adopted.

### 3.2. Characterization of the Sensor

**Figure 8:**
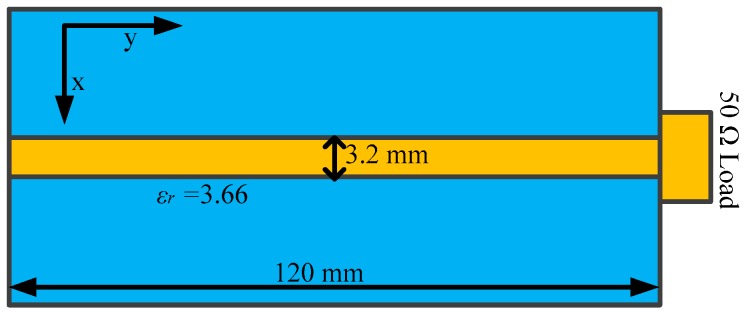
Top view of the characterizing microstrip line.

**Figure 9:**
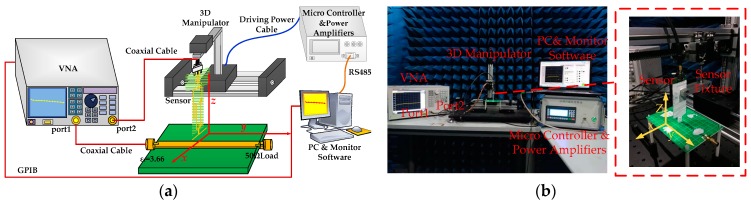
Test setup for characterizing the transmission coefficient of the proposed sensor. (**a**) Schematic diagram and (**b**) photo of the test setup in the half-wave anechoic chamber.

**Figure 10:**
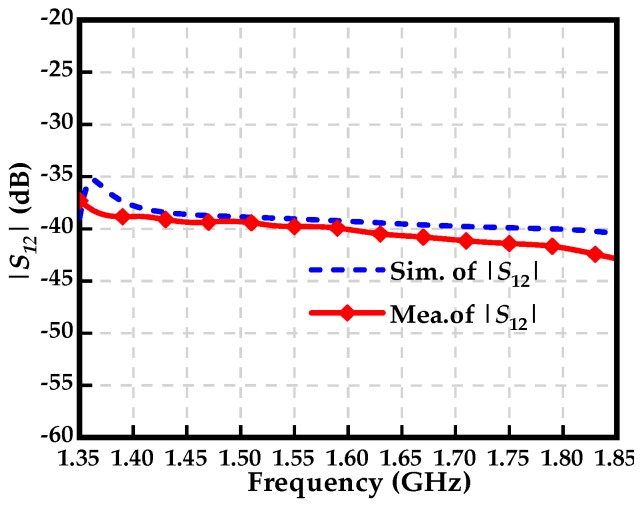
Simulated and measured transmission coefficient of the proposed sensor.

According to the configuration of the measurement system in Figure 7, the characterization of the transmission coefficient for the proposed tangential electric-field sensor is implemented in a half-wave anechoic chamber. A microstrip line, shown in Figure 8, is used as the characterizing source. The characteristic impedance of the microstrip line is 50 Ω. The flatness of the transmission coefficient is an important index for evaluating the designed sensor. It reflects the property of the output frequency response of the sensor with respect to the frequency, i.e., as long as the magnitude of the electromagnetic field is equal, the output voltage of the electric-field sensor should be the same for the electromagnetic wave signal at different frequencies. The microstrip line is terminated with a broadband 50 Ω load so that the travelling wave propagates along the extension direction of the trace. Unlike the characterizations in [26,28], the tangential electric-field sensor is not directly placed 2 mm above the center of the trace of the microstrip line since the maximum tangential electric field is located at a point that has an offset off the trace. Figure 9 shows the test setup for characterizing the transmission coefficient of the proposed sensor. The port 1 of the vector network analyzer drives the microstrip line, and the port 2 acts as a receiver to receive the output of the sensor through a coaxial cable. Figure 10 represents the simulated and measured transmission coefficients. One can see that the simulated result agrees well with the measured one, from 1.35 GHz to 1.85 GHz.

**Figure 11:**
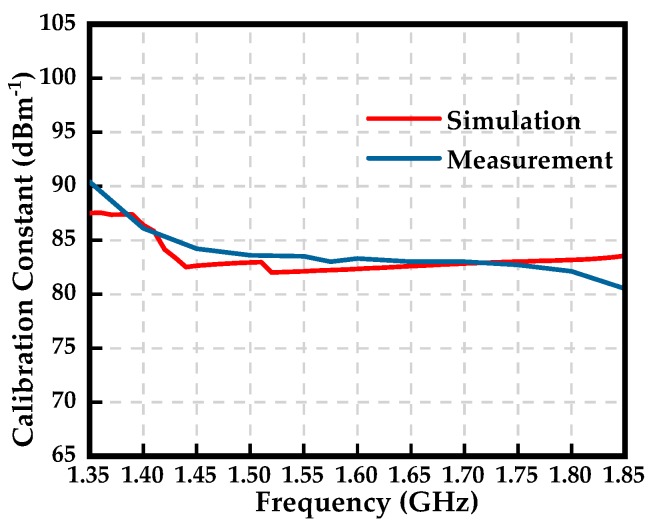
Calibration constant of the proposed sensor. The measurement is obtained with the use of a signal generator and spectrum analyzer.

The calibration constant of the sensor relates the electric field and the output voltage at the test point. The calibration constant of the sensor is defined as the ratio of the measured electric field to the sensor’s output voltage, which is calculated using the following equation.

(3) $$F(f)\left[{\mathrm{dBm}}^{-1}\right]=20\log_{10}(\frac{E}{V})$$

where *V* means the output voltage of the sensor at the receiver port, and *E* means the electric-field strength at the test point. Figure 11 shows the calibration constant of the proposed sensor.

### 3.3. Sensitivity of the Sensor

**Figure 12:**
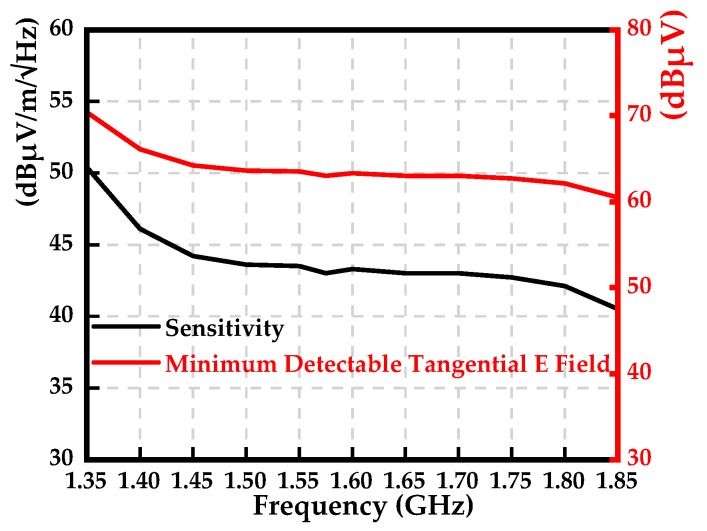
Sensitivity and minimum detectable tangential electric field of the proposed sensor.

The minimum detectable electric field is characterized in terms of the sensitivity of the sensor [20]. The sensitivity of the sensor is related to its output noise and the noise floor of the spectrum analyzer. The designed tangential electric-field sensor in the paper is passive, and the noise floor of the spectrum analyzer is kept unchanged when the sensor is connected. The noise floor of the spectrum analyzer can be regarded as the output noise of the sensor. In this section, the sensitivity of the tangential electric-field sensor is defined in terms of the noise equivalent field strength, which gives a signal-to-noise ratio of 0 dB at the sensor’s output. That sounds a little obscure and not easy to understand. Next, we use an example to present a clear explanation. It is assumed that the resolution bandwidth (RBW) and noise floor of the spectrum analyzer are 100 Hz and −20 dBμV, respectively. Assuming the calibration constant of the sensor is 83 dBm^−1^ at 1.575 GHz, the resulting noise equivalent field strength is 63 dBμV/m (83 dBm^−1^ + (−20 dBμV)), which is regarded as the minimum detectable field strength by the proposed sensor at this frequency. Therefore, the sensitivity of the sensor under the condition of a 100-Hz RBW and −20-dBμV noise floor is 43 dBμV/m/$\sqrt{\mathrm{Hz}}$ (63 dBμV/m/$\sqrt{100\;\mathrm{Hz}}$). Figure 12 shows the sensitivity and minimum detectable tangential electric field of the proposed sensor under the condition of a 100-Hz RBW and −20-dBμV noise floor.

### 3.4. Selectivity of the Sensor

**Figure 13:**
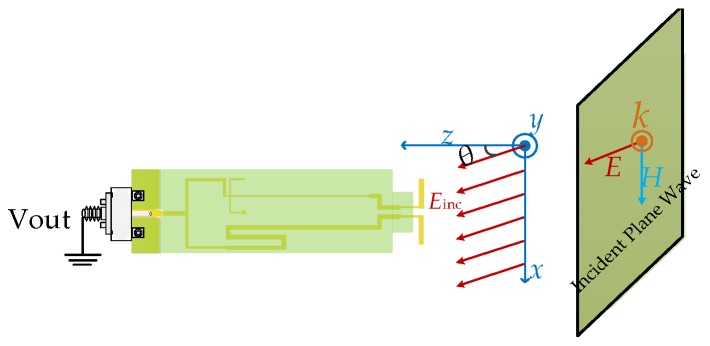
Simulated configuration of the selectivity of the tangential electric-field sensor.

**Figure 14:**
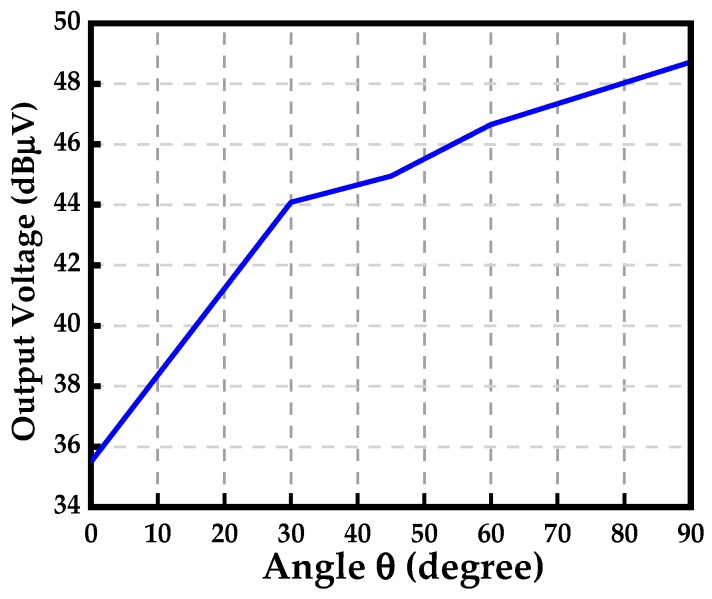
Output voltage of the tangential electric-field sensor illuminated by a plane wave.

Selectivity is also an important characteristic for an electromagnetic sensor, which is often used to characterize antennas and filters. As presented in Section 1, the coaxial monopole (as the sensing part for the electric-field sensor) can only be utilized to sense the surface charge density (normal electric-field component) on the circuit, while the electric dipole can only be used to sense the tangential-field components on a surface over a circuit. It is necessary to investigate the selectivity of the designed tangential electric-field sensor to evaluate the rejection to the normal electric-field. Unfortunately, it is hard to find a pure and standard electric-field source. Therefore, a plane-wave illumination is used as the electric-field excitation. Figure 13 represents the simulated configuration of the selectivity of the tangential electric-field sensor in HFSS. The electric-field vector of the incident plane wave is in an xz-plane, and the angle between the positive direction of the z-axis is θ. Figure 14 shows the simulated output voltage of the tangential electric-field sensor illuminated by a plane wave. The rejection to the normal electric-field (Ez, θ = 90°) of the tangential electric-field sensor is about 14 dB.

### 3.5. Rejection to Magnetic Field (H-Field Rejection)

**Figure 15:**
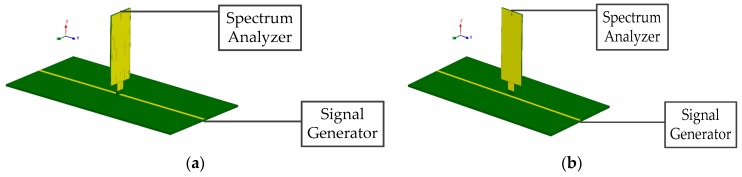
Configuration of the H-field rejection measurement. (**a**) Electric-field coupling and (**b**) magnetic-field coupling. The outputting power of the signal generator is 0 dBm.

**Figure 16:**
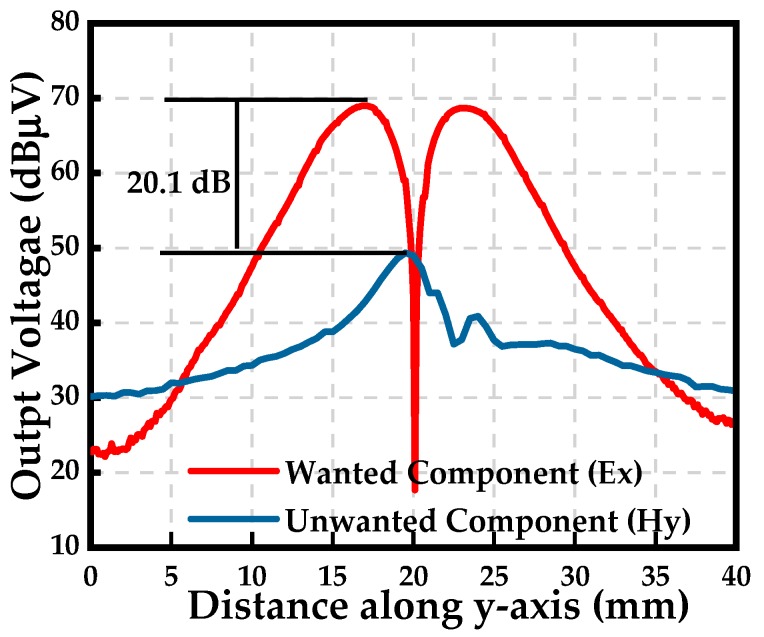
Measured H-field rejection at 1.575 GHz.

Figure 15 shows the configuration of the H-field rejection measurement. The signal generator outputs a stable sinusoidal signal. First, the tangential electric-field sensor is placed above the microstrip line to couple the electric-field component, as is shown in Figure 15a. Then, the tangential electric-field sensor is rotated by 90 degrees around the z-axis to couple the magnetic-field, as is shown in Figure 15b. The tangential electric-field sensor is moved by the measurement system along the x-axis, and the output voltage is recorded. The difference of output voltage of the tangential electric-field sensor in such cases is defined as the rejection to the magnetic field. Figure 16 shows the measured H-field rejection at 1.575 GHz.

### 3.6. Comparisons

Table 3 shows detailed comparisons between the proposed tangential electric-field sensor and the others in order to analysis the performances of the sensor. The physical structure of the proposed sensor is simple and easy to understand, designed for working at other available frequency bands. It can be concluded through the comparisons that it is hard to achieve a single tangential electric-field sensor covering the whole EMC measurement frequency range. The possible reason may be that it is hard to achieve a broadband balun. But there is no doubt that extending the bandwidth of the sensor is keen concern.

## 4. Applications and Validations

### 4.1. Measuring Ex-Field Distribution Over Microstrip Line

**Figure 17:**
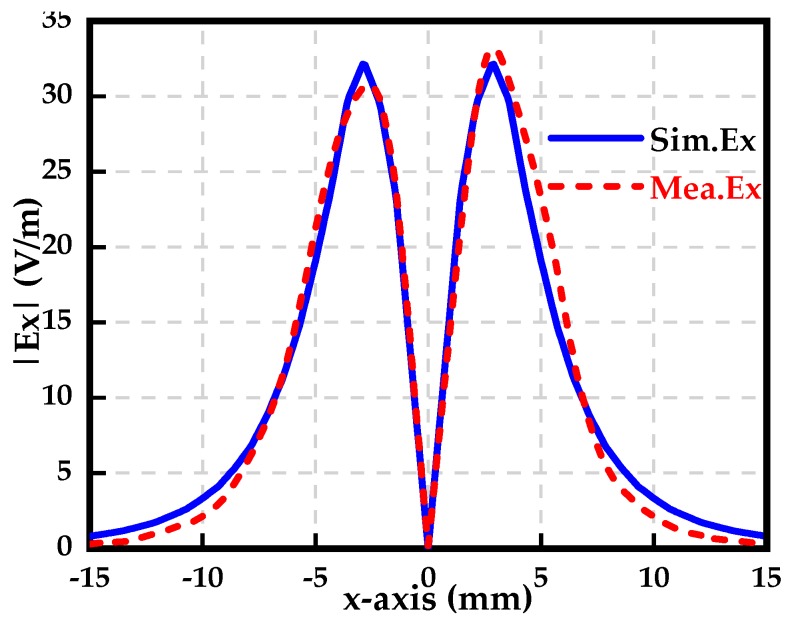
Simulated and measured Ex at the height of 2 mm above the microstrip line.

In order to validate the ability to sense the tangential electric-field component, the microstrip line shown in Figure 8 is used as a DUT. The electromagnetic field generated by the microstrip line can be easily obtained through a full-wave simulation, as the references for the measured values. According to the configurations of the measurement system in Figure 7, a radio frequency (RF) signal generator, outputting a stable sinusoidal signal at a specific frequency, drives the microstrip line. The sensor is placed 2 mm above the microstrip trace, whose center is set as the origin along the x axis. The orientation of the electric dipole arm of the sensor is in the x-axis, since the Ex component dominates for the tangential electric-field component. Figure 17 shows the simulated and measured Ex-field distribution at the height of 2 mm above the microstrip line. It can be seen from Figure 17 that the Ex-field distribution at a specific height above the trace is symmetric along the x-axis to a degree. The two maximum values of the Ex field appear at the two sides respectively, with about a 2.5 mm offset off the center of the trace (*x* = 0), while the minimum value is at *x* = 0 where the field strength is nearly zero.

The simulated Ex field is perfectly symmetric, and the two maximum values have the same magnitude. Additionally, the phase difference between the two maximum values equals 180 degrees. However, the measured Ex field is slightly asymmetric in terms of magnitude. The right maximum value is slightly larger than the left one. The slight difference between the simulation and the measurement is analyzed in [32]. In this paper, the relative error between the simulated field (real field) and the measured field, quantifying the difference, is defined as:

(4) $$E_{\mathrm{error}}=\frac{{\left\|E_{\mathrm{sim}}-E_{\mathrm{mea}}\right\|}_{2}}{{\left\|E_{\mathrm{mea}}\right\|}_{2}}\times 100\%$$

where ${\left\|\right\|}_{2}$ denotes the 2-norm of a vector, which is calculated using:

(5) $${\left\|E_{\mathrm{mea}}\right\|}_{2}=\sqrt{{\sum_{i=1}^{N}\left|E_{\mathrm{mea}}\left(i\right)\right|}^{2}}$$

where i and N mean the index and dimension of the vector, respectively. The calculated relative error is 9.23%, which indicates that the proposed tangential electric-field sensor can competently sense the tangential electric-field component to a degree.

**Figure 18:**
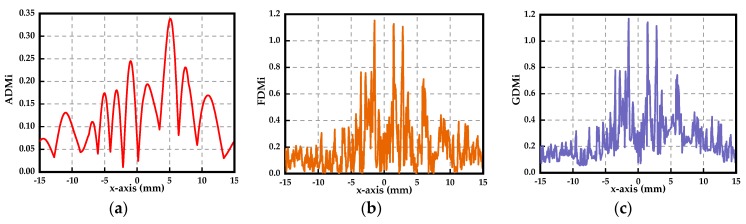
Calculated (**a**) ADM, (**b**) FDM and (**c**) GDM point-by-point comparisons of the data of Figure 17.

**Figure 19:**
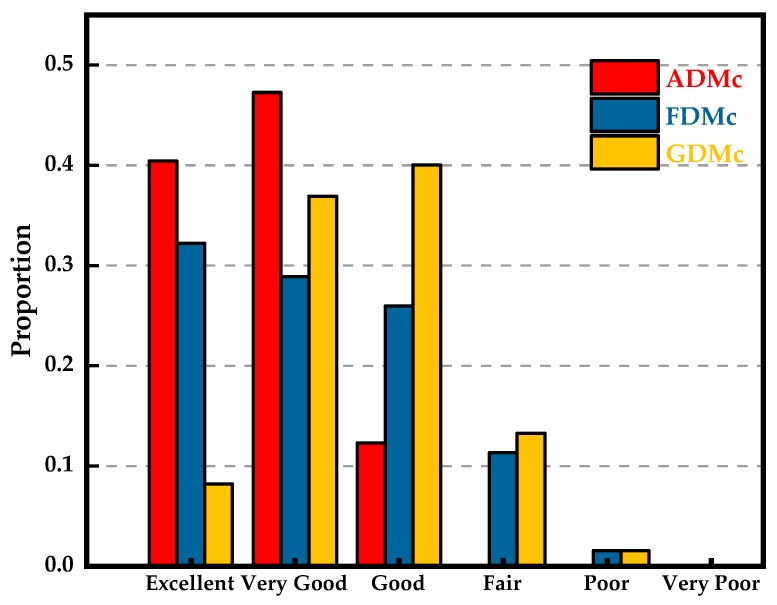
Confidence histograms of the ADM, FDM and GDM obtained from the FSV analysis of the data of Figure 17.

**Figure 20:**
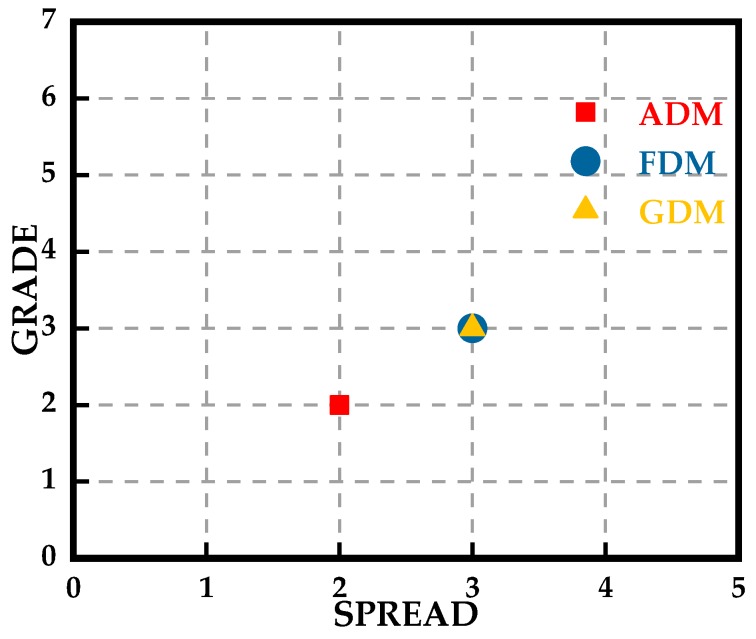
GRADE-SPREAD chart of the data of Figure 17.

The feature selective validation (FSV) method is also introduced in this paper to evaluate the simulated Ex and the measured Ex. The FSV method can quantitatively estimate the comparison of data sets and translate the corresponding compared numerical values to six types of language descriptions (excellent, very good, good, fair, poor, and very poor), giving engineers visual perceptions and more details [44,45]. The amplitude difference measure (ADM) and feature difference measure (FDM) are used to characterize the numerical consistency of the compared data sets in terms of the amplitude and the detailed difference in the feature, respectively. The global difference measure (GDM) is obtained through combining the ADM and FDM. ADMi, FDMi and GDMi are the point-by-point comparisons of the amplitude difference, feature difference, and global difference, respectively. ADMc, FDMc and GDMc represent the density functions that show the proportion of the point-by-point analyses of each of the components with the six types of language descriptions, respectively. The GRADE value is a direct indication of the quality of the comparison, and the SPREAD value indicates the level of reliability of the outputs. We use the FSV method with the help of the FSV Tool [46] to analyze the data of Figure 17. The resulting point-by-point ADM, FDM and GDM are shown in Figure 18. It can be concluded that reducing the quality of the comparisons of the data in Figure 17 is equal to the measured value of Ex at x = 5 mm in terms of the ADM. The serious inconsistency of the simulation and measurement appears at x = 2.5 mm and x = −2.5 mm in terms of the FDM, which agrees well with the intuitive vision that the asymmetry appears at the peak values. The confidence histograms of the data in Figure 17 are shown in Figure 19. The proportions of excellent, very good and good are the main parts, which indicates that the measurement is convincing. The grade-spread chart is also presented in Figure 20. The smaller the GRADE value is, the better the comparison. The smaller the SPREAD value is, the higher the reliability of the results is.

**Figure 21:**
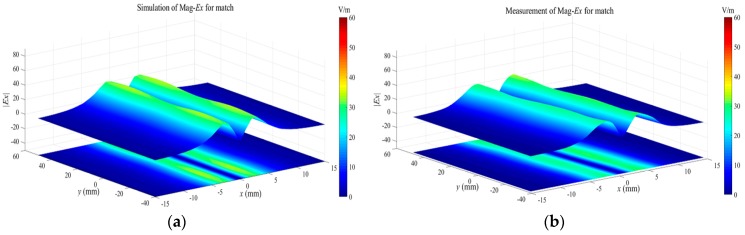
(**a**) The simulated and (**b**) measured Ex-field distributions at the height of 2 mm over the microstrip line in the match state.

**Figure 22:**
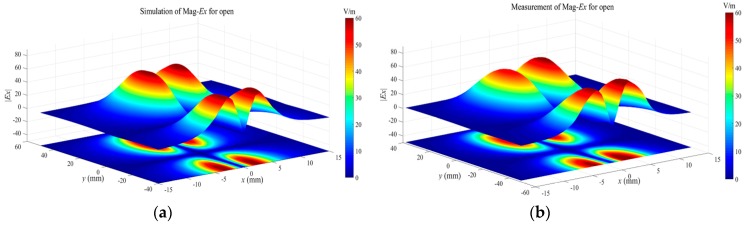
(**a**) The simulated and (**b**) measured Ex-field distributions at the height of 2 mm over the microstrip line in the open state.

**Figure 23:**
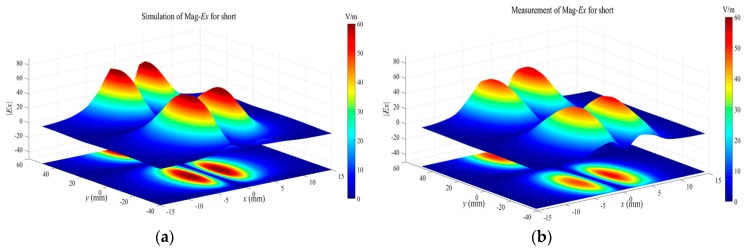
(**a**) The simulated and (**b**) measured Ex-field distributions at the height of 2 mm over the microstrip line in the short state.

Tangential field mappings over the passive microstrip line, operating in three states, are performed to further validate the performance of the proposed tangential electric-field sensor. For the matched microstrip line, the travelling wave propagates along the trace due to the good impedance match. The magnitudes of the sensed tangential electric field should be equal at different positions along the trace. For the open and short microstrip line, the standing wave exists along the trace. The maximum and minimum fields are distributed periodically along the trace. The minimum field, for the short state, dwells where the maximum field exists for the open state. The passive microstrip line is driven by a signal generator (Keysight N5181A) at 1.575 GHz with a power of 0 dBm. The tangential electric-field sensor is placed on the plane 2 mm above the trace, and the orientation of the electric dipole arm of the tangential electric-field sensor is in the *x*-axis to sense the Ex component. The scanning interval along the *x* axis and *y* axis is 1 mm. The scanned data is a big matrix stored in the measurement system. Before the measurements, the simulation works of the field distributions of the microstrip line operating on the three working states are implemented in the full-wave software. The simulated results are shown in Figure 21a, Figure 22a and Figure 23a. As the theoretical analysis shows, the Ex-field distributions over the microstrip line are the standing-wave patterns for the open (Figure 22) and the short (Figure 23). The physical interval between the maximum field and the minimum field is λ/2 for both the open microstrip line and the short microstrip line. There exists a distance shift of λ/4 along the trace for the standing-wave patterns between the open microstrip line and the short microstrip line.

### 4.2. Measuring Tangential E-Field Distribution over Meander Line

**Figure 24:**
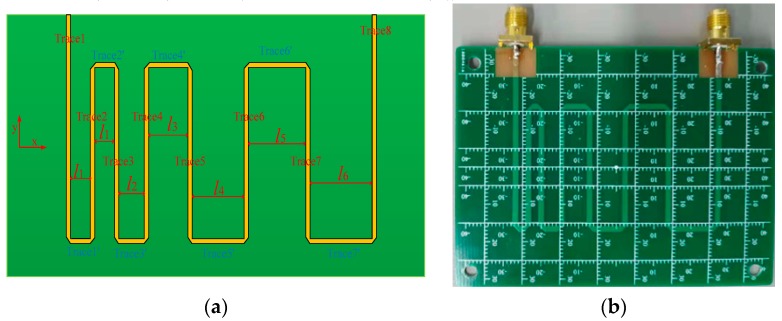
(**a**) Layout of the meander line. *l*_1_ = 2 mm, *l*_2_ = 4 mm, *l*_3_ = 6 mm, *l*_4_ = 8 mm, *l*_5_ = 10 mm, and *l*_6_ = 12 mm. (**b**) Photo of the meander line.

The meander line is a common type of transmission line that is widely used in a high-speed circuit design to adjust the transmission delay of some key signals. The tangential electric field generated by the meander line is investigated by the proposed tangential electric-field sensor. Figure 24 shows the investigated meander line. The gap between each pair of traces increases gradually along the positive direction of the *x*-axis, from 2 mm up to 12 mm, which indicates that the coupling between the adjacent traces decreases, i.e., the coupling between trace 8 and trace 7 is much smaller than that between trace 1 and trace 2.

**Figure 25:**
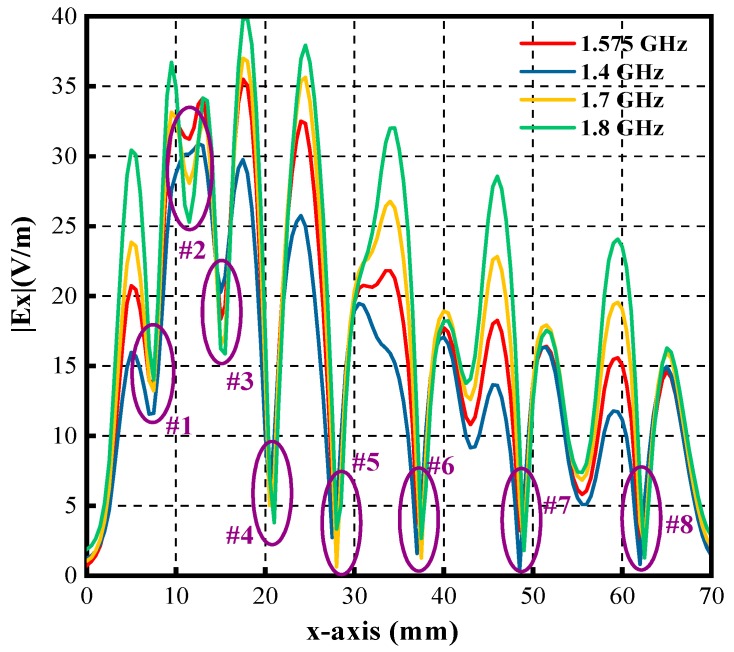
Measured electric field of the meander line along the x-direction.

The experimental results of the Ex-field distribution for the microstrip line in Figure 17 have indicated that the Ex field strength is nearly zero at the center of the trace. The Ex field generated by the meander line is the superposition of several traces. The scanning work is implemented by the measurement system where the instrument is a spectrum analyzer. The scanning length is 70 mm, and the interval is 0.5 mm. The meander line is driven by the signal generator (Keysight N5181A) with a frequency of 1.575 GHz. Figure 25 shows the measured Ex field of the meander line. It is expected that there are 8 “zero-field” positions corresponding to 8 half-width positions. However, only 5 “zero-field” positions are detected in the measurements, and they are marked as #4, #5, #6, #7 and #8 in Figure 25. In theory, the half-width positions of trace 1, trace 2, trace 3, trace 4, trace 5, trace 6, trace 7 and trace 8 correspond to #1, #2, #3, #4, #5, #6, #7 and #8, respectively. Figure 25 indicates that the sensed Ex-field strength is not zero at the positions #1, #2, and #3, where the tangential electric-field sensor fails to distinguish the Ex-field radiation effectively due to the strong coupling from neighboring traces. The reasons for why the spatial resolution of the sensor is not sufficient are analyzed in [33,47]. It can be concluded that the proposed tangential electric-field sensor is not suitable for distinguishing two adjacent traces whose gap is under 4 mm.

**Figure 26:**
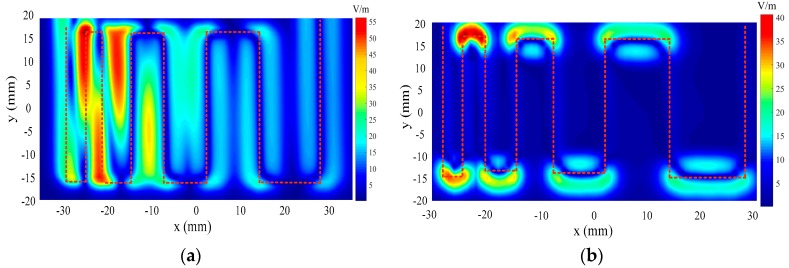
Mappings of the electric-field distribution of the meander line for (**a**) Ex and (**b**) Ey.

The mapping of the tangential electric-field (Ex, Ey) distributions is also conducted to have an insight into the visual tangential electric-field distribution of the meander line. The orientation of the electric dipole arm of the tangential electric-field sensor is parallel to the *x*-axis to pick up Ex. The Ex fields generated by the meander line are mainly contributed by trace 1, trace 2, trace 3, trace 4, trace 5, trace 6, trace 7, and trace 8. From the previous analysis, each trace has a corresponding “zero-field” position along the x-axis. Therefore, a small scanning interval along the x-axis (0.5 mm) is adopted to detect these “zero-field” positions. A coarse scanning interval along the y-axis is set at 1 mm to reduce the dimensions of the scanned data. Figure 26a shows the measured Ex distribution of the meander line. The short dash line indicating the trajectory of the meander line is artificially plotted in Figure 26 to help analyze the result. It is very clearly shown that the Ex field distribution overlaps from trace 2 to trace 3, indicating that the tangential electric-field sensor cannot distinguish these traces well at a close proximity. The tangential electric-field sensor is rotated by 90 degrees around the z-axis so that the orientation of the electric dipole arm of the sensor is parallel to the y-axis to capture the Ey field. Figure 26b validates that the Ey-field radiation generated by the meander line is mainly contributed by trace 1′, trace 2′, trace 3′, trace 4′, trace 5′, trace 6′ and trace 7′. 

### 4.3. Applications in GPS Module

**Figure 27:**
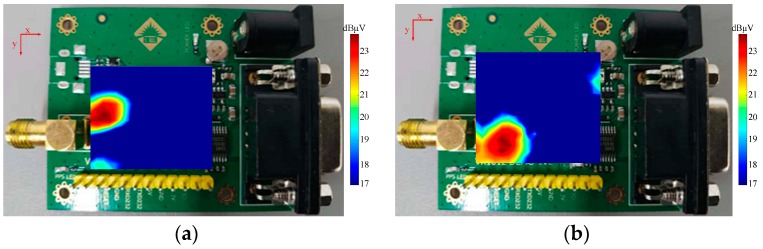
Measured tangential-field distributions of (**a**) Ex and (**b**) Ey, respectively.

An important application of the electromagnetic sensor is to track the coupling trace and to locate the position of the noise source. In this paper, a scanning measurement on a Global Positioning System (GPS) module is conducted. The tangential electric-field sensor is placed as close as possible to the GPS module to enhance the coupling. The scanning interval along the *x*-axis and y-axis is 0.5 mm. The sensor is cascaded with amplifiers to improve the sensitivity. The measured field distributions are shown in Figure 27. The two hot regions are both in the left scanning area. It can be concluded that the working GPS circuit should dwell in the hot region. In the other scanning area, no hot regions are sensed, since the radiation from the GPS module is weak and the sensitivity of the sensor is not high enough.

## 5. Conclusions

In this paper, a tangential electric-field sensor based on a four-layer PCB, embedded with an integrated balun, is designed, manufactured and validated through measurements of passive and active circuits. The electric dipole is the sensed part of the designed sensor for sensing the tangential electric field. The sensed differential-mode voltage is transformed into a common-mode voltage using an integrated balun based on a power diver and phase shifter. Some measurements are conducted to validate the ability of the sensor to pick up the tangential electric field. The performances of the proposed tangential electric-field sensor are also investigated in this paper, such as the sensitivity in terms of the noise equivalent field strength, the selectivity for the tangential electric field and the rejections to other unwanted components.

The applications of the tangential electric-field sensor are presented. First, a microstrip line is measured to validate the accuracy of the sensor when it is used to sense the tangential electric field; the measured results at 1.575 GHz show that the relative error characterizing measurement accuracy is under 10% when the proposed sensor is used to measure the absolute value of the tangential electric field; An FSV analysis technique is also introduced to evaluate the reliability of the measured tangential electric field. Second, the tangential electric fields of the meander line are measured with increasing gaps; the experimental results show that only two adjacent traces with a gap of more than 4 mm are effectively distinguished by the proposed sensor; Finally, the radiations of a real-world GPS module are measured by the sensor. The development of a wideband balun is important for designing a tangential electric-field sensor with a wider bandwidth. Our future work will involve research on the design of a more wideband tangential electric-field sensor.

## Figures and Tables

**Table 1:** Physical dimensions of the designed balun.

$w_{1}$ 0.35 mm	$l_{1}$ 3.00 mm	$w_{1}$ 25.00 mm	$l_{2}$ 49.60 mm
$w_{3}$ 0.21 mm	$l_{3}$ 12.5 mm	$w_{4}$ 2.65 mm	$l_{5}$ 0.35 mm
$l_{4}$ 24.37 mm	$l_{5}$ 6.25 mm	$w_{6}$ 0.11 mm	$l_{6}$ 12.58 mm

**Table 2:** Main parameters of the measurement system.

Motion Distance on x, y, and z Direction	40 mm, 30 mm 30 mm
Mechanical Spatial Resolution	0.1 mm
Supply Voltage of the Power Amplifier	24 V
Version of Micro Controller	TC45 [41]
Communication Protocol	MODBUS 485 Protocol

**Table 3:** Comparisons between the proposed tangential electric-field sensor and the others.

Sensors	Bandwidth	Sensitivity	Spatial Resolution	Balun	Complexity of Structure
This paper	GPS band	High ^1^	4 mm	Passive	Simple
[18]	1 MHz–3 GHz	Low ^1^	Not mentioned	Passive	Complex
[42]	4–8 GHz	Low ^1^	Not mentioned	Passive	Complex
[43]	50 kHz–100 MHz	High ^1^	2 mm ^2^	Active	Complex

^1^ Comparisons of the sensitivity are qualitative, not quantitative. ^2^ It is concluded according to the definitions of the spatial resolution.
